# Therapeutics of the Throat

**Published:** 1887-11

**Authors:** E. B. Nash


### THERAPEUTICS OF THE THROAT.

Editors Homœopathic Physician: You remember my article in the June number of The Homœopathic Physician, entitled "A Proposal for a Work on Therapeutics of the Throat."

At the last meeting of the I. H. A. I interviewed several of the members about it, and these are the names of those who consented to contribute to the work according to my plan, and the remedies they would consent to work up: Adolph Lippe, Lachesis; P. P. Wells, Lycopodium; H. C. Allen, the Mercuries; J. T. Kent, Sulphur; J. B. Bell, Phytolacca; W. P. Wesselhœft, Apis; J. B. Gregg Custis, the Magnesias; W. S. Gee, Bellad.; Wm. A. Hawley, Psorinum.

I inclose you the article on Bellad. already worked by Dr. Gee, also his letter accompanying, as it contains valuable suggestions.

Every member with whom I conferred commended this plan, and, with one or two exceptions, consented to lend a helping hand, but did not decide what particular remedy each would take, except in the names already given. Most of them said it did not make any difference as to that. Now, to expedite this matter and at the same time avoid confusion, may I suggest a few names and the remedy opposite their name that they may work out, if agreeable: J. V. Allen, Ailanthus; Ed. Berridge, Lac. can.; J. A. Biegler, Pulsat.; C. W. Butler, Kali bichrom.; Ed. Carlton, Æsculus hip.; Geo. H. Clark, Ignatia;

Wm. J. Guernsey, Arum. tri.; E. P. Hussey, Nit. acid; Walter M. James, Am. carb.; E. J. Lee, Cistus can.; C. F. Nicols, Sabadilla; A. McNeil, Gelsem. nit.; W. Preston, Alumina; Ed. Rushmore, Argent.; Geo. Sawyer, Capsicum; C. C. Smith, Cantharis.

I only suggest these. There are many more capable men in our I. H. A. and many more remedies not yet mentioned. I may not have suggested the right remedy to the right man. If every one of our members who feel disposed to help on the good work will correspond with me and let me know what he will do, and do it as promptly as possible, like Dr. Gee, we hope to have the work ready for the next meeting of the I. H. A. Now for volunteers.

Who, in addition to remedies already named, will take Causticum, Ars., Baptis., Baryta, Bryon., Hepar sul., Lachnantes, Nux vom., Phos., Rhus tox., Sang., or any other remedy not already taken. There ought to be at least fifty remedies worked up *for all they are worth*, both for acute and chronic diseases of throat. This number, *well done*, with a repertory attached, would make a work of which the I. H. A. might well be proud.

Let every one be sure to have his or her part ready by January 1st, 1888. That will give time for the repertory.

All I desire to do is to have the privilege (with the suggestions of my brethren) of doing the *preface*. Dr. Brown has consented to write a short article on the hygiene of throat. We know him to be capable.

Now, this is downright work; and every one should give his best and most careful effort to make his or her contribution reliable. No theorizing. Facts are what we want, so that when we turn to the work of our Society on therapeutics of the throat we may feel sure we are referring to the best work extant.

As these remedies come out in the journals there will be plenty of chance for kindly criticism and additional hints from all their readers.

If we can thus concentrate the thought and labor and collect the experience and observation of the membership of such a Society as ours, upon a single subject so important as this one, it seems as though great good must come out of it. Now, cannot this letter, with that of Dr. Gee's and his article on Belladonna, come out in the next number of the PHYSICIAN, so as to get the subject early before the Society? We hope so.

This is everybody's work, and each may have a part in the labor and a share in the honors and the rewards.

Yours, fraternally,

 E. B. NASH.

